# Supplementary material for: Systematic screening of viral and human genetic variation identifies antiretroviral resistance and immune escape link
Source: eLife. 2021 Jun 1;10:e67388. doi: 10.7554/eLife.67388 (PMC8169104; doi:10.7554/eLife.67388)
Supplement: Supplementary file 1. — Overview of the Benjamini–Hochberg procedure to correct for multiple testing in selecting HLA–mutation pairs. Pairs were ranked by the p-value of the HLA term in the adjusted logistic regression model predicting for the queried mutation. The numerical rank (I) is divided by the total number of pairs (m = 225) and multiplied by the false discovery rate of 0.2 (Q). With this adjustment, the lowest-ranked pairs where the p-value is lower than (I/m)Q, along with all pairs ranked above, are included after the adjustment (in bold in the table), yielding the three candidate pairs we investigated in-depth (in bold). Only the first 25 rows of the total 225 are shown. [file elife-67388-supp1.docx]

| DRM:HLA Pair | Rank (I) | P value | (I/m)Q |
| --- | --- | --- | --- |
| **RT-E138:HLA-B18** | **1** | **0.000000** | **0.000889** |
| **RT-E138:HLA-A24** | **2** | **0.000021** | **0.001778** |
| **RT-V179:HLA-B35** | **3** | **0.001162** | **0.002667** |
| RT**-**V106**:**HLA-A32 | 4 | 0.023042 | 0.003556 |
| RT**-**V179**:**HLA-C04 | 5 | 0.042533 | 0.004444 |
| RT**-**E138**:**HLA-B44 | 6 | 0.046104 | 0.005333 |
| RT**-**E138**:**HLA-C07 | 7 | 0.048058 | 0.006222 |
| RT**-**E138**:**HLA-A11 | 8 | 0.059825 | 0.007111 |
| RT**-**V179**:**HLA-A03 | 9 | 0.061083 | 0.008000 |
| RT**-**L210**:**HLA-C07 | 10 | 0.068847 | 0.008889 |
| RT**-**E138**:**HLA-C14 | 11 | 0.076254 | 0.009778 |
| RT**-**V179**:**HLA-C16 | 12 | 0.081507 | 0.010667 |
| RT**-**E138**:**HLA-C12 | 13 | 0.081528 | 0.011556 |
| RT**-**K219**:**HLA-C07 | 14 | 0.082888 | 0.012444 |
| PR**-**M46**:**HLA-B35 | 15 | 0.083177 | 0.013333 |
| RT**-**E138**:**HLA-A23 | 16 | 0.084422 | 0.014222 |
| RT**-**T215**:**HLA-B15 | 17 | 0.091116 | 0.015111 |
| RT**-**K103**:**HLA-B51 | 18 | 0.106090 | 0.016000 |
| RT**-**V106**:**HLA-B40 | 19 | 0.111217 | 0.016889 |
| RT**-**M184**:**HLA-A02 | 20 | 0.112279 | 0.017778 |
| PR**-**L90**:**HLA-C04 | 21 | 0.112902 | 0.018667 |
| RT**-**V179**:**HLA-B27 | 22 | 0.112908 | 0.019556 |
| PR**-**M46**:**HLA-C07 | 23 | 0.115195 | 0.020444 |
| RT**-**E138**:**HLA-A32 | 24 | 0.125272 | 0.021333 |
| PR**-**L33**:**HLA-C04 | 25 | 0.131358 | 0.022222 |
