# Supplementary material for: Systematic screening of viral and human genetic variation identifies antiretroviral resistance and immune escape link
Source: eLife. 2021 Jun 1;10:e67388. doi: 10.7554/eLife.67388 (PMC8169104; doi:10.7554/eLife.67388)
Supplement: Supplementary file 2. — For each HLA–mutation pair, the binding peptides (defined as below a rank of 2% for weakly binding and below 0.5% for strongly binding) are listed ranked in decreasing predicted binding strength according to NetMHCpan. Peptides in bold denote the peptides without the mutation that bind more strongly than all other peptides for that position in the viral amino acid sequence. Peptides in bold and italics denote peptides without the mutation that bind more weakly than a mutated form. [file elife-67388-supp2.docx]

| DRM:HLA Pair | Peptide | Score | Rank (%) |
| --- | --- | --- | --- |
| RT-E138:HLA-B18 | **NETPGIRYQ** | **0.1036** | **0.77** |
|  | NNKTPGIRY | 0.085387 | 0.89 |
|  | NNATPGIRY | 0.080966 | 0.93 |
|  | **ETPGIRYQY** | **0.044507** | **1.4** |
|  | ***NNETPGIRY*** | ***0.042344*** | ***1.4*** |
|  | NNGTPGIRY | 0.027694 | 1.8 |
| RT-E138:HLA-A24 | KTPGIRYQY | 0.064341 | 1.2 |
|  | ATPGIRYQY | 0.035626 | 1.6 |
| RT-V179:HLA-B35 | **NPDIVIYQY** | **0.987276** | **0.01** |
|  | NPDIEIYQY | 0.979827 | 0.01 |
|  | NPDIDIYQY | 0.977098 | 0.01 |
|  | **NPDIFIYQY** | **0.975329** | **0.01** |
|  | KQNPDIVIY | 0.198012 | 0.62 |
|  | KQNPDIFIY | 0.172234 | 0.68 |
|  | KQNPDIEIY | 0.123943 | 0.89 |
|  | KQNPDIDIY | 0.074717 | 1.3 |
