# Supplementary material for: Systematic screening of viral and human genetic variation identifies antiretroviral resistance and immune escape link
Source: eLife. 2021 Jun 1;10:e67388. doi: 10.7554/eLife.67388 (PMC8169104; doi:10.7554/eLife.67388)
Supplement: Transparent reporting form [file elife-67388-transrepform.pdf]

Westbrook Centre
Milton Road
Cambridge CB4 1YG, UK

P 01223 855340
W elifesciences.org
T @elife

eLife's transparent reporting form

We encourage authors to provide detailed information *within their submission* to facilitate the interpretation and replication of experiments. Authors can upload supporting documentation to indicate the use of appropriate reporting guidelines for health-related research (see EQUATOR Network), life science research (see the BioSharing Information Resource), or the ARRIVE guidelines for reporting work involving animal research. Where applicable, authors should refer to any relevant reporting standards documents in this form.

If you have any questions, please consult our Journal Policies and/or contact us: editorial@elifesciences.org.

Sample-size estimation

- You should state whether an appropriate sample size was computed when the study was being designed
- You should state the statistical method of sample size computation and any required assumptions
- If no explicit power analysis was used, you should describe how you decided what sample (replicate) size (number) to use

Please outline where this information can be found within the submission (e.g., sections or figure legends), or explain why this information doesn't apply to your submission:

As this study analysed cohort data to screen for DRM:HLA pairs with an association suggesting a selective pressure, we did not perform sample-size estimations in advance. However, in the Materials and Methods, in the "SCREENING CANDIDATE PAIRS OF DRM:HLA-TYPE" section, we do describe how power calculations were used to include or exclude candidate pairs based on whether or not there were sufficient numbers to find an association.

Replicates

- You should report how often each experiment was performed
- You should include a definition of biological versus technical replication
- The data obtained should be provided and sufficient information should be provided to indicate the number of independent biological and/or technical replicates
- If you encountered any outliers, you should describe how these were handled
- Criteria for exclusion/inclusion of data should be clearly stated
- High-throughput sequence data should be uploaded before submission, with a private link for reviewers provided (these are available from both GEO and ArrayExpress)

Please outline where this information can be found within the submission (e.g., sections or figure legends), or explain why this information doesn't apply to your submission:

(Due to the nature of the cohort study data, discussion on replicates is not applicable.)

Inclusion/exclusion criteria are outlined in Figure 1 and Table 1.

Sequence data used for the analyses do not fulfil the requirements for open data access, due to Swiss legal constraints and the informed consent obtained for the study participants.

eLife Sciences Publications, Ltd is a limited liability non-profit non-stock corporation incorporated in the State of Delaware, USA, with company number 5030732, and is registered in the UK with company number FC030576 and branch number BR015634 at the address Westbrook Centre, Milton Road, Cambridge CB4 1YG.

Statistical reporting

- Statistical analysis methods should be described and justified
- Raw data should be presented in figures whenever informative to do so (typically when N per group is less than 10)
- For each experiment, you should identify the statistical tests used, exact values of N, definitions of center, methods of multiple test correction, and dispersion and precision measures (e.g., mean, median, SD, SEM, confidence intervals; and, for the major substantive results, a measure of effect size (e.g., Pearson's r, Cohen's d)
- Report exact p-values wherever possible alongside the summary statistics and 95% confidence intervals. These should be reported for all key questions and not only when the p-value is less than 0.05.

Please outline where this information can be found within the submission (e.g., sections or figure legends), or explain why this information doesn't apply to your submission:

> The statistical methods used are detailed in the Materials and Methods, in the "SCREENING CANDIDATE PAIRS OF DRM:HLA-TYPE" section. Figure 1 and Table 1 and their legends state the breakdown of the study population. Figures 2 and 3 detail all the relevant information for the statistical analyses performed. The results of the Benjamini Hochberg adjustment are shown in Supplementary Table S1.

(For large datasets, or papers with a very large number of statistical tests, you may upload a single table file with tests, Ns, etc., with reference to sections in the manuscript.)

Group allocation

- Indicate how samples were allocated into experimental groups (in the case of clinical studies, please specify allocation to treatment method); if randomization was used, please also state if restricted randomization was applied
- Indicate if masking was used during group allocation, data collection and/or data analysis

Please outline where this information can be found within the submission (e.g., sections or figure legends), or explain why this information doesn't apply to your submission:

> (Not applicable, as this is observational cohort data and there was no group allocation.)

Additional data files ("source data")

- We encourage you to upload relevant additional data files, such as numerical data that are represented as a graph in a figure, or as a summary table
- Where provided, these should be in the most useful format, and they can be uploaded as "Source data" files linked to a main figure or table
- Include model definition files including the full list of parameters used
- Include code used for data analysis (e.g., R, MatLab)
- Avoid stating that data files are "available upon request"

Please indicate the figures or tables for which source data files have been provided:

eLife Sciences Publications, Ltd is a limited liability non-profit non-stock corporation incorporated in the State of Delaware, USA, with company number 5030732, and is registered in the UK with company number FC030576 and branch number BR015634 at the address Westbrook Centre, Milton Road, Cambridge CB4 1YG.

The individual-level datasets generated and analyzed for the current study do not fullfill the requirements for open data access: (1) The SHCS informed consent states that sharing data outside the SHCS network is only permitted for specific studies on HIV infection and its related complications, and to researchers who have signed an agreement detailing the use of the data and biological samples; and (2) the data is too dense and comprehensive to preserve patient privacy in persons living with HIV.

Per Swiss law, data cannot be shared if data subjects have not agreed or if data is too sensitive to share. Investigators with a request for the data that support the findings of this study should contact the corresponding author Roger Kouyos and the Scientific Board of the SHCS. The provision of data will be considered by the Scientific Board of the SHCS and the study team and is subject to Swiss legal and ethical regulations, and is outlined in a material and data transfer agreement.

We have however, provided the datafiles (with the rows anonymized and randomly re-assorted) with the bare minimum number of variables necessary to assemble the figures as shown in the manuscript.

The code for the analysis can be found on Github repository:
https://github.com/hnyhnyhny/HNGUYEN_HLA_DRM

eLife Sciences Publications, Ltd is a limited liability non-profit non-stock corporation incorporated in the State of Delaware, USA, with company number 5030732, and is registered in the UK with company number FC030576 and branch number BR015634 at the address Westbrook Centre, Milton Road, Cambridge CB4 1YG.
